# Ancestral self-compatibility facilitates the establishment of allopolyploids in Brassicaceae

**DOI:** 10.1007/s00497-022-00451-6

**Published:** 2022-10-25

**Authors:** Polina Yu. Novikova, Uliana K. Kolesnikova, Alison Dawn Scott

**Affiliations:** Department of Chromosome Biology, Max Planck Institute for Plant Breeding Research, Carl-von-Linne-Weg 10, 50829 Cologne, Germany

**Keywords:** Self-incompatibility, Polyploidy, S-locus, Brassicaceae

## Abstract

Self-incompatibility systems based on self-recognition evolved in hermaphroditic plants to maintain genetic variation of offspring and mitigate inbreeding depression. Despite these benefits in diploid plants, for polyploids who often face a scarcity of mating partners, self-incompatibility can thwart reproduction. In contrast, self-*compatibility* provides an immediate advantage: a route to reproductive viability. Thus, diploid selfing lineages may facilitate the formation of new allopolyploid species. Here, we describe the mechanism of establishment of at least four allopolyploid species in Brassicaceae (*Arabidopsis suecica*, *Arabidopsis kamchatica, Capsella bursa-pastoris,* and *Brassica napus*), in a manner dependent on the prior loss of the self-incompatibility mechanism in one of the ancestors. In each case, the degraded *S*-locus from one parental lineage was dominant over the functional *S*-locus of the outcrossing parental lineage. Such dominant loss-of-function mutations promote an immediate transition to selfing in allopolyploids and may facilitate their establishment.

## Links between selfing and polyploidy

Polyploids are organisms with more than two complete sets of chromosomes, resulting from whole-genome duplication within one lineage (autopolyploids) or between different lineages (allopolyploids). All extant diploid plants are ancient polyploids (Masterson 1994), and about 30% of flowering plants are recent polyploids (neopolyploids) with relatively equal contributions of auto- and allo-origins (Wood et al. 2009; Barker et al. 2016). Despite the ubiquity of this phenomenon, newly formed polyploids are rarely successful over evolutionary timescales (Mayrose et al. 2011), as many factors can impede their survival.

The first reproductive challenge of new polyploids is faithful segregation of doubled chromosomes during meiosis (Bomblies et al. 2015, 2016), which may require genetic adaptation in both auto- and allopolyploids. Specific variants in genes mediating synapsis (which reduce recombination between homologous chromosomes) seem to be under strong selection in autopolyploids (Yant et al. 2013; Bray et al. 2020; Morgan et al. 2020; Seear et al. 2020; Bohutínská et al. 2021), while genes reducing homoeologous exchanges are selected for in allopolyploids (Riley and Chapman 1958; Sears 1977; Jenczewski et al. 2003; Henry et al. 2014; Burns et al. 2021).

**Fig. 1:**
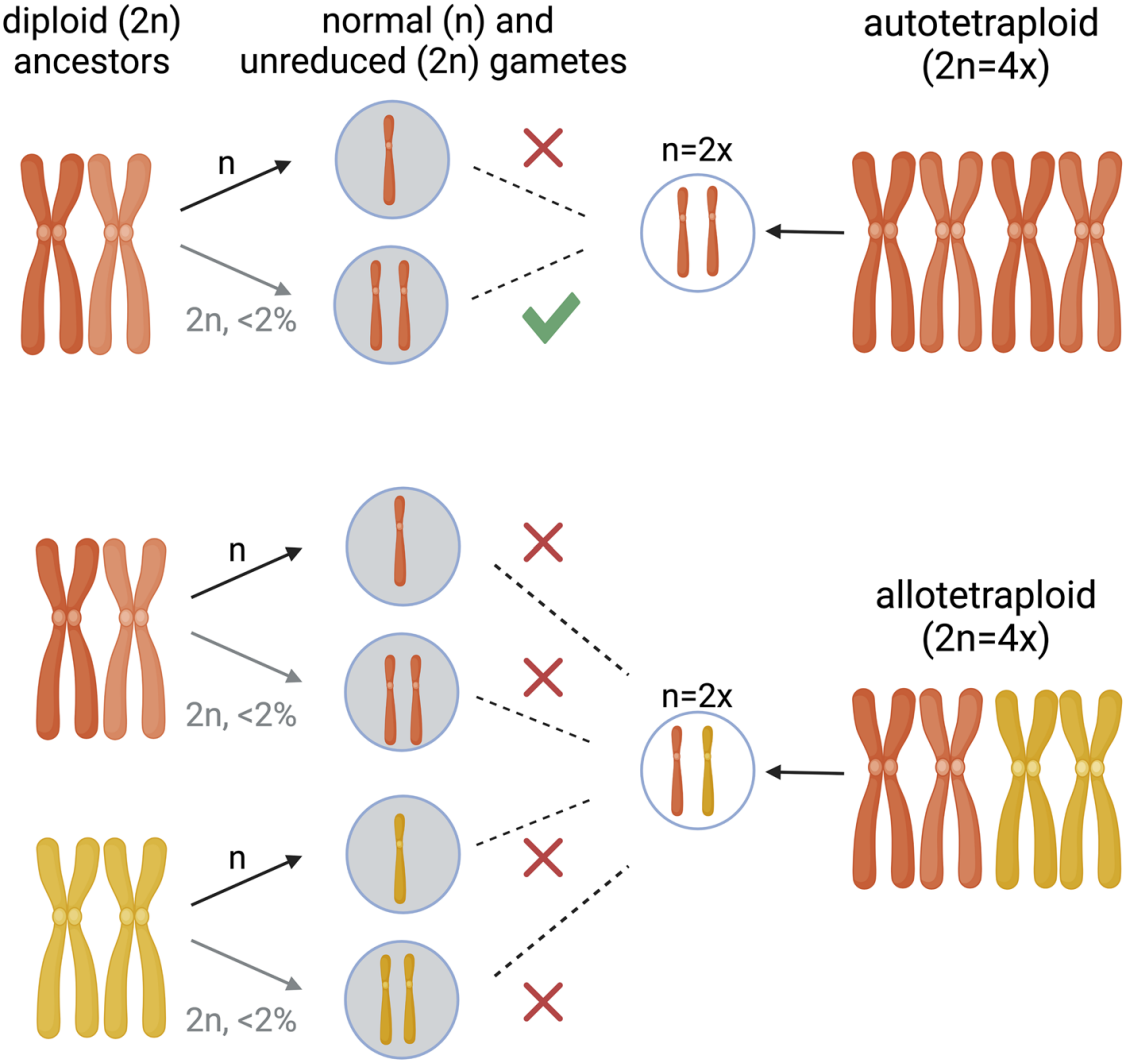
Newly formed polyploids often lack mating partners with compatible karyotypes. While autotetraploids are compatible with rare (< 2% in Brassicaceae on average (Kreiner et al. 2017b)) unreduced gametes from the diploid ancestral populations, allotetraploids are not. Immediate transition to self-compatibility in allotetraploids could facilitate their establishment

The scarcity or complete absence of mating partners with compatible karyotypes is another challenge for emerging polyploids (Levin 1975). While new autopolyploid individuals are compatible with the 2*n* gametes of their diploid progenitors, new allopolyploids may require self-compatibility to propagate sexually (Fig. 1). Additionally, reproductive competition with diploids, which are present in larger numbers, can limit the availability of mating partners for new polyploids (Levin 1975). Although the overall association between selfing and polyploidy is relatively weak (Mable 2004), there are stronger correlations between specific types of genetically based self-incompatibilities and different types of polyploids (Mable 2004; Barringer 2007; Husband et al. 2008). The formation of a stably reproducing population from newly originated rare polyploids can be promoted by a transition to self-fertilization (Shimizu et al. 2004). While a transition to selfing in diploids often leads to inbreeding depression when deleterious recessive mutations are exposed in homozygotes, in polyploids the negative impacts of selfing may be alleviated by additional allelic copies that mask recessive mutations and maintain high fitness (Lande and Schemske 1985; Comai 2005; Rosche et al. 2017).

Any polyploid originates from a cell cycle abnormality, either meiotic, leading to unreduced gametes, or mitotic, leading to somatic doubling. A polyploid individual can directly form as a result of two unreduced gametes merging, or in following generations via a so-called “triploid bridge” after the merging of an unreduced gamete with a haploid gamete. A diploid plant with a somatically doubled meristem can also form 2n (or higher ploidy) gametes or even give rise to polyploid seeds if the plant is self-compatible. Various transitions from diploidy to polyploidy were reviewed by Comai (2005). While unreduced gametes are considered the primary cause of polyploidy (Thompson and Lumaret 1992; Bretagnolle and Thompson 1995; Kreiner et al. 2017a) and the evidence for somatic doubling is scarce (Newton and Pellew 1929; Nasrallah et al. 2007; Bachmann et al. 2021), the relative contribution of each route to polyploid formation is unknown.

Apart from aiding the establishment of polyploids, selfing can also have a direct effect on their formation by promoting unreduced (2*n*) gamete formation (Kreiner et al. 2017b), which complicates inference of the causality between self-compatibility and whole-genome duplications. In natural plant populations, the rate of 2*n* gamete formation is usually lower than 2%, but is highly variable, with many factors affecting the rate (e.g. reproductive mode and life history) (Kreiner et al. 2017b). For example, selfing plants experience lower selection pressure on correct meiotic outcomes and thus have higher rates of unreduced gametes (Kreiner et al. 2017b), which suggests yet another way selfing can promote the origin of polyploids. At the same time, 2*n* gametes are experimentally inducible under stress conditions (De Storme et al. 2012; Mason and Pires 2015; Zhou et al. 2015), such as extreme temperature (Mason et al. 2011; Mason and Pires 2015; Zhou et al. 2015). This may explain the association of natural polyploids with harsh environments (Vanneste et al. 2014; Lohaus and Van de Peer 2016; Van de Peer et al. 2017; Novikova et al. 2018), although a putative adaptive advantage of polyploids could drive this association.

## Types of genetic self-incompatibility and association with polyploidy

One way to classify self-incompatibility in plants is based on the genetics underlying pollen phenotype. In the so-called gametophytic self-incompatibility type, the phenotype of pollen is determined by its own haploid genome. In the sporophytic self-incompatibility type, the phenotype of pollen is determined by the diploid anther genome. The prevailing “gametophytic type” is an umbrella term for at least two mechanistically different systems, one characteristic of Solanaceae, Rosaceae, and Scrophulariaceae, based on the S-RNase degradation of pollen tubes, the other found in Papaveraceae, where pollen tube growth is inhibited by Ca^2+^ influx. For comprehensive reviews of the evolution and mechanisms of different self-incompatibility types, please see (Silva and Goring 2001; Takayama and Isogai 2005; Charlesworth et al. 2005).

Interestingly, in the Solanaceae-like gametophytic self-incompatibility system, polyploidization itself can automatically lead to selfing (Entani et al. 1999; Takayama and Isogai 2005; Robertson et al. 2011; Zenil-Ferguson et al. 2019). In this case, self-incompatibility in diploids is based on a heterozygous (S_1_S_2_) pistil expressing S_1_ and S_2_ cytotoxic S-RNases which are both taken up by haploid pollen (S_1_ or S_2_) that are only able to inhibit non-self S-RNases. In tetraploids (S_1_S_1_S_2_S_2_), homozygous pollen (S_1_S_1_ or S_2_S_2_) is also rejected, while a heterozygous pollen (S_1_S_2_) can inhibit both types of S-RNases and is therefore compatible (McClure 2009). In the other gametophytic Papaveraceae-like systems, the association between polyploidy and selfing is not as strong and there is no evidence that whole-genome duplications can cause self-incompatibility loss (Mable 2004; Paape et al. 2011). Families with sporophytic self-incompatibility system also did not show a strong association between polyploidy and selfing (Mable 2004). Based on the few known examples from the Brassicaceae family it seems that while autopolyploids can maintain an ancestral outcrossing mating type (Dart et al. 2004; Hollister et al. 2012; Hohmann et al. 2014; Novikova et al. 2016, 2018; Melichárková et al. 2020), a transition to selfing is more common in allopolyploids and probably aids their establishment (Okamoto et al. 2007; Tsuchimatsu et al. 2012; Kitashiba and Nasrallah 2014; Novikova et al. 2017; Akiyama et al. 2020; Bachmann et al. 2021; Kolesnikova et al. 2022). The majority of polyploid Brassicaceae are lacking either information on origin (allo vs auto) and/or mating system (self-compatible vs self-incompatible), not to mention the genotypes underlying mating types. We thus focus our review on the few allotetraploid Brassicaceae species with known *S*-locus genotypes and draw parallels in the genetic mechanism of their transition to selfing.

## Sporophytic self-incompatibility in Brassicaceae

**Fig. 2:**
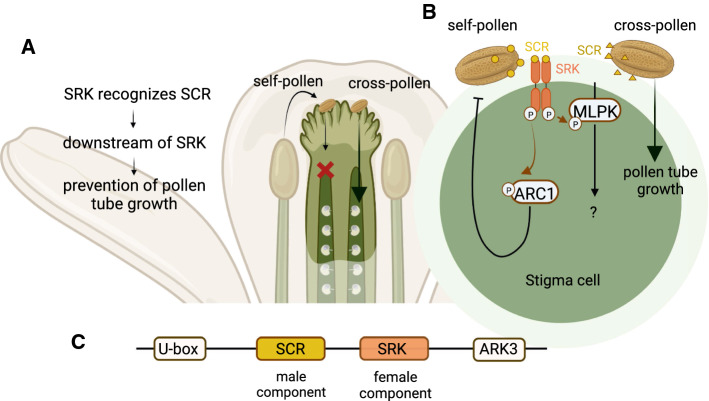
The mechanism of the sporophytic self-incompatibility system, typical for the Brassicaceae family. **a** In a self-pollination attempt, the SRK receptor on the surface of the pistil will recognize the SCR ligand on the surface of pollen which will switch on a downstream signalling cascade leading to the prevention of pollen tube growth. **b** Recognition of SCR ligand by SRK receptor results in formation of a heterotetrameric structure (Ma et al. 2016) and induces autophosphorylation of SRK. ARC1 (arm repeat containing 1) and MLPK (M-locus protein kinase) interact with SRK and positively regulate the downstream reaction, as knockout of these genes can also lead to self-compatibility (Chen et al. 2019). ARC1 is an E3 ubiquitin ligase which activates proteasomal protein degradation (Stone et al. 2003; Samuel et al. 2008). Although *Arabidopsis*/*Capsella* and *Brassica* self-incompatibility systems share the same major players, some differences also exist (Yamamoto and Nishio 2014). For example, in *Brassica* SLG (S-locus glycoprotein) protein is also present in stigma, which enhances the self-incompatibility reaction upon SCR-SRK recognition (Takayama et al. 2001). **c** A schematic representation of the *S*-locus: genes coding for male (*SCR* in Arabidopsis or *SCR/SP11* in Brassica) and female (*SRK*) components are strongly linked together, forming a haplotype typically flanked by *U-box (U-box/ARM repeat protein* or *B80)* and *ARK3* (*receptor kinase 3*) genes (Kusaba et al. 2001; Hagenblad et al. 2006). *SCR* will be recognized as “self” by *SRK* from the same haplotype

Sporophytic self-incompatibility in Brassicaceae is based on the recognition between the pistil receptor (SRK, S receptor kinase) and pollen ligand (SP11/SCR, *S*-locus protein 11/*S*-locus cysteine-rich protein), which initiates a kinase cascade inhibiting pollen tube growth involving autophagy (Fig. 2) (Suzuki et al. 1999; Schopfer et al. 1999; Takayama et al. 2000; Takasaki et al. 2000; Kusaba et al. 2001; Macgregor et al. 2022). SRK/SCR based self-incompatibility is ancient and most probably ancestral to all Brassicaceae (Fobis-Loisy et al. 2004). SRK is a membrane protein with the extracellular domain reacting with short SCR ligand, the trans-membrane domain passing the signal, and the cytoplasmic domain with protein kinase activity (Stein et al. 1991; Takayama et al. 2001). Both SCR and SRK proteins have conserved cysteines which are structurally important and for recognition function (Watanabe et al. 1994; Kusaba et al. 2001; Mishima et al. 2003); loss of function in either SCR or SRK leads to the breakdown of self-incompatibility (Goring et al. 1993; Nasrallah et al. 1994; Tsuchimatsu et al. 2010). The term *sporophytic* means that both *SCR* and *SRK* genes are expressed in the sporophytic (2*n*) cells: the *SRK* gene is expressed in the same papilla cell where the protein is localized, while *SCR* (or *SCR/SP11*) is expressed in tapetum cells of anthers, and the protein is secreted and then embedded into the pollen coat (Schopfer et al. 1999; Takayama et al. 2000).

Self-recognition genes are multiallelic and extremely diverse, sharing high proportion of polymorphism between species and even between genera (Schierup et al. 2001; Castric and Vekemans 2007). *S*-alleles are trans-specifically shared between the genera *Arabidopsis, Crucihimalaya* and *Capsella* (Schierup et al. 1998; Paetsch et al. 2006; Castric and Vekemans 2007; Busch et al. 2008; Tedder et al. 2011; Guo et al. 2011; Leducq et al. 2014; Zhang et al. 2019), which had a common ancestor about 11–14 million years ago (Hohmann et al. 2015; Mandáková et al. 2017). However, while *Leavenworthia alabamica* evolved a secondary non-syntenic *S*-locus (Busch et al. 2008, 2011; Chantha et al. 2013, 2017), its close relative from the same tribe, *Cardamine hirsuta*, has a colinear *S*-locus to Arabidopsis and Brassica and the S-haplogroup of selfing *C. hirsuta* is orthologous to *A. halleri* and *A. lyrata S*-allele from S-haplogroup 1 (Gan et al. 2016). This suggests that *S*-alleles are probably shared even between Arabidopsis and Cardamine, which have diverged about 18–22 Mya (Hohmann et al. 2015; Mandáková et al. 2017).

The reason for such long-standing shared variation is the active maintenance of *S*-allele diversity in outcrossing populations by frequency-dependent balancing selection (Wright 1939; Vekemans and Slatkin 1994; Mable et al. 2003; Castric and Vekemans 2004; Kamau and Charlesworth 2005; Castric et al. 2008; Llaurens et al. 2008; Roux et al. 2013): a rare allele has more chances to propagate, while a more common allele has a higher risk to be falsely recognized as “self”. Both *SRK* and *SCR* genes are linked by suppressed recombination in the *S*-locus which leads to their co-evolution in highly divergent S-haplotypes (Nasrallah 2005; Guo et al. 2011; Goubet et al. 2012). Outcrossing populations typically have 10–35 segregating *S*-alleles (Castric and Vekemans 2004) which ensures their reproductive success. Outstanding diversity of *S*-alleles complicates studying of self-incompatibility (Mable et al. 2018) and new *S*-locus alleles are still being discovered with increased availability of sequencing data and improved analytical tools (Genete et al. 2020). Several excellent reviews describe our current understanding of the sporophytic self-incompatibility mechanism (Takayama and Isogai 2005; Fujii and Takayama 2018; Jany et al. 2019; Nasrallah 2019; Durand et al. 2020); here, we only highlight the relevant features known to play a role in immediate breakdown of self-incompatibility in allotetraploids.

## Dominance mediated self-compatibility in Brassicaceae hybrids.

*S*-alleles can be dominant, co-dominant, or recessive and the dominance relationships can differ in pollen and stigma (Bateman 1954). *SRK* alleles in Brassicaceae are often co-dominant, so in heterozygous individuals both alleles of *SRK* are expressed (Hatakeyama et al. 2001; Kusaba et al. 2002; Prigoda et al. 2005; Okamoto et al. 2007), while in *SCR/SP11* co-dominance is rare and usually only one allele is expressed (Llaurens et al. 2008; Schoen and Busch 2009; Fujii and Takayama 2018). Pollen-based (*SCR*) dominance is more well-characterized than pistil-based (*SRK*) dominance, and is based on a *trans*-acting silencing mechanism (Tarutani et al. 2010; Durand et al. 2014). Comparison of a few dominant and recessive alleles demonstrated that a dominant *S*-allele produces microRNAs which can silence expression of *SCR* on a recessive *S*-allele, which possess specific targets for the microRNAs (Tarutani et al. 2010; Durand et al. 2014; Fujii and Takayama 2018). Silencing is achieved through methylation of a 5’ promoter sequence of *SCR* on a recessive *S*-allele (Kusaba et al. 2002; Shiba et al. 2006). Such dominance is gradual: the more recessive the *S*-allele, the more targets for microRNAs from different *S*-alleles it has (Durand et al. 2014), meaning more opportunity to be silenced. The dominance hierarchy of *S*-alleles also appears to be shared between species, as precursors of microRNAs and their targets are tightly linked to specific S-haplotypes by suppressed recombination (Tarutani et al. 2010; Durand et al. 2014).

**Fig. 3:**
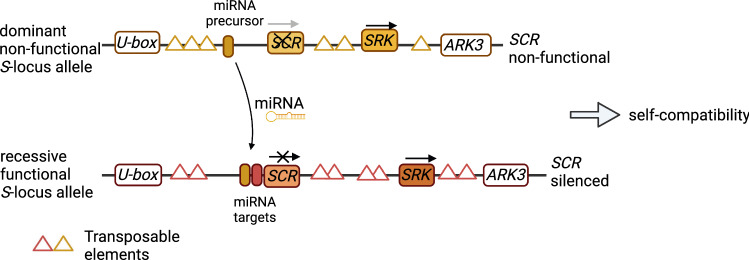
Schematic representation of an immediate transition to selfing in a heterozygous individual with a loss-of-function *S*-allele dominant in anthers. Dominant *S*-alleles carry precursors that produce microRNAs with targets on the recessive *S*-alleles. Such cross talk allows the dominant *S*-allele to silence *SCR* expression on the recessive *S*-allele, regardless of the functionality of the *SCR* on the dominant *S*-allele. Same mechanism applies for allotetraploids with non-allelic (homoeologous) *S*-loci on different subgenomes. When a dominant *S*-locus homeolog in the allotetraploid is inherited from a selfing species, it is non-functional in terms of self-recognition but can silence a functional *S*-locus homeolog inherited from outcrossing species, leading to immediate self-incompatibility breakdown

Although the genetics underlying the self-recognition function and the described dominance/recessiveness characteristics are both linked to the *S*-locus, they are uncoupled from each other. For example, an *S*-allele can lose self-recognition function but remain dominant. Therefore, heterozygous individuals with one non-functional *S*-allele can remain self-incompatible if the *S*-allele is recessive or co-dominant, or can become self-compatible if the *S*-allele with broken self-recognition is dominant (Fig. 3). As *S*-alleles are shared between species and the dominance mechanism acts in *trans* (Mable et al. 2004; Tarutani et al. 2010; Durand et al. 2014), *S*-alleles appear to interact similarly in heterozygous diploids, interspecific hybrids, and natural allopolyploids (Nasrallah et al. 2007; Okamoto et al. 2007; Tsuchimatsu et al. 2012; Kitashiba and Nasrallah 2014; Novikova et al. 2017; Bachmann et al. 2021) (Fig. 3).

The epigenetic mechanism of self-incompatibility breakdown in interspecific hybrids has been shown in the F_1_ progeny of *A. thaliana* x *A. lyrata* and *C. rubella* x *C. grandiflora* crosses (Nasrallah et al. 2007). *A. thaliana* and *C. rubella* are selfing species, while *A. lyrata* and *C. grandiflora* are outcrossing. Stigmas of the F_1_ hybrids resulted from a *A. thaliana* x *A. lyrata* cross were functional but failed to recognize parental *A. lyrata* pollen in a backcross, thus allowing pollen tube growth. The loss of self-incompatibility on stigmas of *A. thaliana* x *A. lyrata* hybrids was linked to aberrant splicing of *SRK* gene transcripts (Nasrallah et al. 2007). In these experiments, F_1_ *A. thaliana* x *A. lyrata* hybrids failed to produce pollen and were effectively male-sterile due to unmatched chromosome numbers in parental genomes (*n* = 5 and *n* = 8 respectively). However, somatic mutation on one of the F_1_ *A. thaliana* x *A. lyrata* hybrids produced neo-allopolyploids, which restored normal meiosis, produced functional pollen, and were self-fertile (Nasrallah et al. 2007).

In contrast to *A. thaliana* × *A. lyrata* crosses, the cross between *C. rubella* and *C. grandiflora* produced fertile F_1_ hybrids, as parental species have the same numbers of chromosomes (*n* = 8). In the F_2_ population of selfed F_1_ hybrids, self-compatibility segregated as a single-locus, dominant trait: plants homozygous for the *S*-allele inherited from *C. rubella* were self-compatible; homozygous plants from *C. grandiflora* were self-incompatible, and heterozygous plants were self-compatible. However, in this case, self-compatibility was linked to the loss of expression of *SCR* and ultimately pollen-driven (Nasrallah et al. 2007). Thus, at least two epigenetic mechanisms can lead to loss of self-incompatibility in hybrids: splicing errors of *SRK* transcripts in stigmas and downregulation of *SCR* in anthers.

## Immediate transition to self-compatibility in Brassicaceae allotetraploids.

In allopolyploid species, *S*-locus homeologs on different subgenomes interact in the same manner as S-alleles in heterozygous diploids and previously described amphidiploid hybrids (Nasrallah et al. 2007; Okamoto et al. 2007; Tsuchimatsu et al. 2012; Kitashiba and Nasrallah 2014; Novikova et al. 2017; Bachmann et al. 2021) (Fig. 3). If a de novo mutation leading to a loss of the self-recognition function occurs on a dominant *S*-locus homeolog, or such a mutation inherited from a selfing progenitor with a dominant *S*-allele, this will lead to an immediate transition to selfing (Fig. 3). To our knowledge, there are no described natural allotetraploid species in Brassicaceae that are obligate outcrossers with a fully retained self-incompatibility system. Immediate transition to selfing after an interspecific cross where one parent carries a dominant loss-of-function mutation in the S-locus can mitigate one of the first challenges on the path to allotetraploid establishment, i.e. a lack of the compatible mating partners (Fig. 1). Below, we describe four known cases where such a mechanism led to the transition to selfing in allopolyploids, thus facilitating their establishment (Table 1).

**Table 1:** Examples of Brassicaceae allotetraploid species where selfing is determined by a loss-of-function mutation in the dominant *S*-allele

Allotetraploid	Maternal species, S-alleles		Paternal species, S-alleles		Dominance hierarchy in known S-allele combinations	Source
*A. suecica*	*A. thaliana* (SC)	AthS-A (AhS4), SCR non-functional	*A. arenosa* (SI)	AarS17 (AhS2)	AhS4 > AhS2 (anther based)	Novikova et al. (2017)
*A. kamchatica*	*A. halleri* (SI)	AkS-A (AhS26), AkS-B (AhS47), AkS-C (AhS1)	*A. lyrata* (SI/SC)	AkS-D (AhS12), SCR non-functional; AkS-E (AhS2)	AhS12 > AhS1 (anther based); AhS26 = /? AhS12 (codominant in pistil); AhS47 ? AhS12; AhS47 > AhS2 (anther based)	Tsuchimatsu et al. (2012), Kolesnikova et al. (2022)
*C. bursa-pastoris*	*C. orientalis* (SC)	CbpS-B (AhS12), SCR non-functional; CbpS-? (SRR8904462)	A common ancestor of *C. rubella* and *C. grandiflora* (SI)	CbpS-A (AhS64/AhS62)	AhS12 > AhS64/AhS62 (anther based)	Bachmann et al. (2019, 2021)
*B. napus*	*B. rapa* (SI)	class I: BnS-1 (BrS-47, SCR/SP11 non-functional), BnS2 (BrS-21), BnS-3 (BrS-8), BnS-4 (BoS-13), BnS-5 (BrS-32); class II: BnS-7( BrS-29)	*B. oleracea* (SI)	BnS-6 (class II, BoS-15)	BnS-1 > BnS-6 (anther based); BnS-2 > BnS-6 (pistil based); BnS-3 > BnS-6 (pistil based)	Okamoto et al. (2007), Kitashiba and Nasrallah (2014)

For easier comparison, we show *A. halleri* orthologs of *S*-alleles for Arabidopsis and Capsella species in brackets

*SI* self-incompatible;* SC* self-compatible. For known combinations of *S*-alleles in the allotetraploids, we use “ > ” sign to show more dominant allele on the left, “ = ” to show co-dominant alleles, and “?” sign in cases where dominance hierarchy is not known

### *Arabidopsis suecica*

Allotetraploid species *A. suecica* (2*n* = *4x* = 26) originated ~ 16 Kya from a hybridization between maternal plant *A. thaliana* (2*n* = 10) and paternal *A. arenosa* (2*n* = 16 or 2*n* = 4*x* = 32), likely arising in Central Europe but with a current distribution in the Fennoscandian region (Hylander 1957; Price et al. 1994; Mummenhoff 1995; Sall et al. 2003; Säll et al. 2004; Jakobsson et al. 2006; Novikova et al. 2017). Based on chloroplast divergence estimations, the two parental lineages (*A. arenosa* and *A. thaliana*) diverged roughly 6 Mya (Hohmann et al. 2015; Mandáková et al. 2017). Demographic inferences based on whole genome population genetics (The 1001 Genomes Consortium 2016; Durvasula et al. 2017; Fulgione et al. 2018; Fulgione and Hancock 2018) and divergence times at *S*-alleles (Bechsgaard et al. 2006; Shimizu et al. 2008) concur and together suggest that the *A. thaliana* lineage migrated to North Africa ~ 1 Mya, where it transitioned to selfing and experienced a karyotypic change from eight chromosomes (2*n* = 16) to five (2*n* = 10) around 500 Kya. Subsequently, *A. thaliana* spread all over the Northern Hemisphere after the last glaciation maximum peaked at 20Kya (Beck et al. 2008; François et al. 2008; The 1001 Genomes Consortium 2016; Lee et al. 2017; Hsu et al. 2019).

To date, it is unclear whether a diploid or autotetraploid lineage of *A. arenosa* gave rise to allopolyploid *A. suecica*. Both diploid and autotetraploid lineages of *A. arenosa* are obligate outcrossers, suggesting that *A. suecica* inherited a functional *S*-allele from *A. arenosa*. The diversity of *S*-alleles in *A. thaliana* has been reduced to four non-functional haplogroups (A, B, C, and a recombinant one between A and C), which co-occur all together only in North Africa (Durvasula et al. 2017). Although *A. suecica* originated from multiple individual crosses, it inherited only one *S*-allele from *A. thaliana* and one from *A. arenosa * (Novikova et al. 2017). From *A. thaliana*, *A. suecica* inherited *S*-allele from S-haplogroup A where an ancestral 213-bp inversion in the *SCR* gene led to the loss of self-recognition in ancestral species (Tsuchimatsu et al. 2010). This *A. thaliana S*-allele is orthologous to *A. halleri S*-allele 4 (AhS4). The *A. suecica S*-allele that *A. suecica* inherited from *A. arenosa* is orthologous to *A. halleri S*-allele 2 (AhS2) (Novikova et al. 2017). Using a series of controlled crosses in *A. halleri*, it was shown that AhS4 allele is dominant over AhS2 in pollen and co-dominant in pistil (Llaurens et al. 2008). The pollen-based dominance is explained by expression of mir867 from AhS4 which is able to target the first exon of AhS2 *SCR* gene (Durand et al. 2014). Both the microRNA precursor (mir867) and its target were shown to be conserved in *A. suecica S*-alleles inherited from *A. thaliana* and *A. arenosa* respectively (Novikova et al. 2017). Together this suggests that *A. suecica* could transition to self-compatibility immediately after the cross between *A. thaliana* with a dominant but non-functional *S*-allele (AhS4 with broken *SCR* gene) and *A. arenosa* with a recessive but functional *S*-allele (AhS2).

### *Arabidopsis kamchatica*

*A. kamchatica* is an allotetraploid species that originated from hybridization between *A. lyrata* and *A. halleri* in East Asia (Shimizu et al. 2005; Shimizu-Inatsugi et al. 2009). Multiple haplotypes in *A. kamchatica* chloroplasts and *S*-loci suggest multiple founding hybridization events in this lineage. All of the four different chloroplast haplotypes were *A. halleri*-derived, suggesting that *A. halleri*, and not *A. lyrata*, always served as a maternal lineage (Shimizu-Inatsugi et al. 2009; Tsuchimatsu et al. 2012). *A. halleri* is an obligate outcrosser with a fragmented geographical range in Europe and in East Asia (Al-Shehbaz and O’Kane 2002). *A. lyrata* is predominantly outcrossing with two independently originated selfing lineages: an older one in Siberia (~ 140 Kya) with a wide distribution from Taymir to Chukotka (Paape et al. 2018; Kolesnikova et al. 2022), and a younger one (~ 10 Kya) in North America occurring around the Great Lakes region (Mable et al. 2005; Foxe et al. 2010; Griffin and Willi 2014; Carleial et al. 2017). *A. halleri subsp. gemmifera* from East Asia and Siberian selfing *A. lyrata* are genetically the closest lineages to *A. kamchatica* (Shimizu et al. 2005; Shimizu-Inatsugi et al. 2009; Paape et al. 2018; Kolesnikova et al. 2022). Demographic modelling based on spectra of neutral variants from the two *A. kamchatica* subgenomes estimated the divergence time of the hybrid species from the ancestral *A. halleri* in the range of ~ 87–105 Kya and *A. lyrata* in the range of 121–145 Kya (Paape et al. 2018).

The transition to selfing in Siberian *A. lyrata* is most probably associated with a self-incompatibility breakdown in a single individual as all of the found selfing populations in Siberia shared the same *S*-allele closest to *A. halleri* allele S12 (Kolesnikova et al. 2022). Three *S*-alleles segregate in the *A. halleri*-derived subgenome (AkS-A, AkS-B and AkS-C) and two *S*-alleles segregate in the *A. lyrata*-derived subgenome (AkS-D and AkS-E) of *A. kamchatica*, among which the AkS-D (orthologous to *A. halleri* S12), inherited from the Siberian selfing *A. lyrata* (Kolesnikova et al. 2022), is the most frequently observed (Tsuchimatsu et al. 2012). Some of the *A. kamchatica* accessions bearing AkS-D (AhS12) *S*-alleles showed incompatible reactions in pistils when crossed with pollen from *A. halleri* with orthologous *S*-alleles, suggesting that *SRK* gene on the AkS-D (AhS12) is functional (Tsuchimatsu et al. 2012). Siberian selfing *A. lyrata* accessions either completely lost *SCR* gene or lost one of the conserved cysteines important for structural integrity of the SCR protein (Kolesnikova et al. 2022). Together this suggests that the loss of self-incompatibility in Siberian selfing *A. lyrata* is most probably male-driven and one of the *S*-alleles that *A. kamchatica* inherited from *A. lyrata* (AkS-D/AhS12) had unfunctional *SCR*. All three combinations of homeologs with AkS-D/AhS12 S-allele on the *A. lyrata* subgenome and AkS-A/AhS26, AkS-B/AhS47 or AkS-C/AhS1 on *A. halleri* subgenome of *A. kamchatica* are possible and have been shown in the population data (Tsuchimatsu et al. 2012). *A. halleri S*-allele S12 is predicted to be pistil-dominant over S1 allele as S12 contains the microRNA precursor sequence *mirS3*, which may silence *SCR* gene expression on S1 allele (Llaurens et al. 2008; Durand et al. 2014). The *S*-locus of selfing Siberian *A. lyrata* also contains *mirS3* sequence (Kolesnikova et al. 2022). This suggests that in the combination with *A. halleri* S1 homeolog (AkSRK-C) in *A. kamchatica*, *A. lyrata* homeolog will silence *SCR* expression of *A. halleri* homeolog and turn *A. kamchatica* with this combination of *S*-alleles into a self-compatible plant in the first generation (Table 1). The potential mechanism of self-incompatibility breakdown in *A. kamchatica* with other combinations of *S*-locus homeologs is less clear (Table 1).

Although geographic distributions of *A. halleri* and *A. lyrata*, close relatives of *A. kamchatica* progenitors, also overlap in Europe (Clauss and Koch 2006; Schmickl et al. 2010) and crosses between *A. halleri* and *A. lyrata* in Europe do not display any obvious genetic incompatibilities (Sarret et al. 2009), the allotetraploid *A. kamchatica* formed only in East Asia. As selfing *A. lyrata* populations were found only in Siberia and North America and not in Europe (Mable et al. 2005; Foxe et al. 2010; Hu et al. 2011; Griffin and Willi 2014; Kolesnikova et al. 2022), this suggests that the possibility of forming a self-compatible hybrid in East Asia facilitated the establishment of allopolyploid *A. kamchatica* in this region.

### *Capsella bursa-pastoris*

*Capsella* and *Arabidopsis* diverged 8–10 Mya and belong to the same lineage I clade of Brassicaceae (Hohmann et al. 2015; Mandáková et al. 2017). Of the three diploid species in *Capsella*, two are self-compatible (*C. rubella* and *C. orientalis*) and one is an obligate outcrosser (*C. grandiflora*) (Guo et al. 2009; Hurka et al. 2012). Currently, *C. orientalis* is distributed in central Asia, while *C. rubella* and *C. grandiflora* are generally restricted to the European continent. Despite their present distribution, their ranges overlapped in the past, as they hybridized to form an allotetraploid *Capsella bursa-pastoris* about 200–300 Kya via hybridization between selfing *C. orientalis* and a common ancestor of *C. rubella* and *C. grandiflora* (Douglas et al. 2015; Kasianov et al. 2017). Speciation of *C. rubella* from the obligate outcrosser *C. grandiflora* is more recent, about 30–50 Kya, and associated with a transition to self-compatibility in a single individual (Guo et al. 2009; Slotte et al. 2013; Koenig et al. 2019).

Analysis of chloroplast sequences of Capsella genus showed that maternal contribution to the allotetraploid *C. bursa-pastoris* came from *C. orientalis * (Hurka et al. 2012; Omelchenko et al. 2020*)*. Extensive haplotype sharing between *C. orientalis* and *C. bursa-pastoris* suggested that ancestral *C. orientalis* was highly homozygous and therefore already selfing when it contributed to *C. bursa-pastoris* (Douglas et al. 2015). Transition to selfing in *C. orientalis* is associated with a single frame-shift deletion in the *SCR* gene, which was found to be fixed across 32 *C. orientalis* samples from 18 populations (Bachmann et al. 2019). All the *C. orientalis* samples shared the same *S*-allele. Because selfing in *C. orientalis* is associated with a single *S*-allele, the timing of the transition could be estimated based on accumulated polymorphisms in the *S*-locus since then. The time boundaries for the self-incompatibility loss in *C. orientalis* was estimated by calculating time to the most recent common ancestor between orthologous *S*-alleles of *C. orientalis* and *C. bursa-pastoris* for the lower boundary which amounted to 70 Kya and between *C. orientalis* and *C. grandiflora* for the upper boundary which amounted to 2.6 Mya (Bachmann et al. 2019), which is much older compared to *C. rubella*. In crosses between *C. orientalis* and *C. grandiflora*, self-compatibility mapped to the *S*-locus as a dominant trait (Bachmann et al. 2019). Similarly, orthologous to *C. orientalis S*-allele, *A. halleri* allele S12 in crosses between different *A. halleri* accessions was dominant (Llaurens et al. 2008). This shows once again that not only are *S*-alleles trans-specifically shared, but also their dominance hierarchy appears to be conserved across *Arabidopsis* and even *Capsella*.

The population structure of *C. bursa-pastoris* suggests that its multiple origins span distinct geographical regions, such as Europe, the Middle East, and Asia (Cornille et al. 2016; Kryvokhyzha et al. 2016; Wesse et al. 2021). However, in the subgenome inherited from *C. orientalis,* all but one *C. bursa-pastoris* accession from putatively distinct origins share the same frameshift deletion in *SCR* with *C. orientalis* (Bachmann et al. 2019, 2021), consistent with the notion that *C. orientalis* was probably selfing long before it contributed to the allotetraploid *C. bursa-pastoris* (Douglas et al. 2015). The only *C. bursa-pastoris* accession with a different *S*-allele in the *C. orientalis* subgenome was sampled in Central Asia (Cbp_DUB-RUS9, accession number SRR8904462 (Kryvokhyzha et al. 2019)). In the other subgenome (inherited from an ancestor of *C. rubella* and *C. grandiflora*) all the C. *bursa-pastoris* accessions shared the same *S*-allele, orthologous to *A. lyrata* S38 and S30 (Bachmann et al. 2021). Importantly, the *S*-locus homeolog in the *C. orientalis* subgenome of *C. bursa-pastoris* (orthologous to *A. halleri* S12) retained the microRNA precursor mirS3 with target on the *S*-locus homeolog in the other subgenome (Durand et al. 2014; Burghgraeve et al. 2020; Bachmann et al. 2021). Due to the challenges of *SCR* annotation (in part because of its small size, structure, and high diversity), it was not possible to assess the impact of *mirS3* on *SCR* expression from the *C. rubella*/*C. grandiflora* subgenome of *C. bursa-pastoris*. However, it is likely that a dominant *S*-locus allele with a non-functional *SCR* inherited from *C. orientalis* can downregulate a functional *SCR S*-locus allele inherited from *C. rubella*/*C. grandiflora* and render the hybrid immediately self-compatible.

### *Brassica napus*

In *Brassica*, roughly 50 different trans-specifically shared *S*-alleles were identified (Nou et al. 1993; Ockendon 2000) and classified as either class I or class II based on their dominance levels (Nasrallah et al. 1991; Nasrallah and Nasrallah 1993). In heterozygous individuals with class I and class II alleles, only class I alleles are expressed (Hatakeyama et al. 1998). A separate dominance hierarchy also exists within class II (Kakizaki et al. 2003; Shiba et al. 2006). A single mutation in a dominant *S*-allele can induce self-compatibility in agriculturally important *Brassica* crops, as demonstrated in *B. napus* (Goring et al. 1993; Silva et al. 2001; Okamoto et al. 2007).

All the diploid Brassica species from the Triangle of U—*B. rapa* (AA, 2*n* = 20), *B. nigra* (BB, 2*n* = 16) and *B. oleracea* (CC, 2*n* = 18)—are self-incompatible, while the natural allotetraploids, *B. juncea* (AABB, 2*n* = 4*x* = 36), *B. napus* (AACC, 2*n* = *4x* = 38), and *B. carinata* (BBCC, 2*n* = 4x = 34), are all self-compatible species (Nagaharu 1935). Natural allotetraploid *B. napus* originated about 7.5 Kya in the Mediterranean region (Chalhoub et al. 2014). Chloroplast analysis found three different haplotypes suggesting multiple origin of B. napus with *B. rapa* as a maternal parent in a cross with *B. oleracea* (Allender and King 2010). The parental species, *B. rapa* and *B. oleracea*, are highly diverse at the *S*-locus with 30 and 50 *S*-alleles, respectively (Nou et al. 1993; Ockendon 2000), while the allotetraploid *B. napus* has only seven *S*-alleles (Okamoto et al. 2007): five from dominant class I (BnS-1–5) and two from recessive class II (BnS-6–7). The parental origin of some *S*-alleles is unclear, but genotyping and segregation analysis of F_2_ populations suggested that *B. napus* is fixed for the *B. oleracea*-inherited recessive BnS-6 allele and the remainder of the *S*-alleles segregate in the *B. rapa*-inherited subgenome (Okamoto et al. 2007). The fact that *B. napus* has inherited only one allele from *B. oleracea* does not contradict the possibility of multiple origins, as recessive alleles are usually most frequent. This is because recessive alleles are effectively hiding from being recognized and rejected which leads to their higher frequency in the population (Schierup et al. 1997; Billiard et al. 2007; Genete et al. 2020).

The most frequent *S*-allele combination in *B. napus* is *B. rapa*-derived BnS-1 and *B. oleracea*-derived BnS-6. BnS-1 was shown to be pollen-dominant, meaning it can suppress the *SCR/SP11* gene expression on BnS-6 allele. At the same time, BnS-1 exhibits a disruptive insertion in the promoter region of its own *SCR/SP11* gene, so self-compatibility in *B. napus* with BnS-1/BnS-6 *S*-alleles is explained by the fact that it does not express any *SCR/SP11* (Okamoto et al. 2007). For *B. napus* individuals with two additional combinations of *S*-alleles (BnS-2/BnS-6 and BnS-3/BnS-6), self-compatibility was explained by disruptive mutations in *SRK* of the stigma-dominant (BnS2 and BnS3) alleles (Okamoto et al. 2007).

In contrast to the natural allotetraploid *B. napus*, lab crosses between the diploid *B. rapa* and *B. oleracea* did not result in self-compatible progeny (Nishi 1968; Tsunoda et al. 1980; Beschorner et al. 1995). Moreover, such resynthesized *B. napus* allopolyploids often show genomic instability resulting from homoeologous exchanges during meiosis (Xiong et al. 2021; Ferreira de Carvalho et al. 2021). There is a growing interest in the production of fertile and stable allopolyploid Brassica hybrids to enrich the gene pool of existent crops and create new allopolyploid crops (Xiao et al. 2019; Hu et al. 2019; Zhang et al. 2021). Identifying *S*-alleles that can ensure immediate self-compatibility in such neo-allopolyploids can contribute to the production of fertile and agronomically important genetic combinations.

## Conclusion

The origin and establishment of an allopolyploid species requires a whole series of happy coincidences, which we describe as its evolutionary history. These requisites include geographical overlap between parental species and appropriate environmental conditions for hybridization and subsequent survival of the new hybrid. In Brassicaceae, the right combinations of *S*-alleles leading to an immediately self-fertile hybrid progeny also seem to be a crucial condition on the road to establishing an allopolyploid. Transition to selfing at the very origin of the allotetraploids can be achieved if one of the parental species is already selfing and this loss of self-compatibility is associated with a mutation in the dominant *S*-allele. This is a tight constraint given considerable negative genomic consequences of selfing in diploid (potential ancestral) populations and the fact that dominant *S*-alleles are relatively rare. Together, this may explain why allotetraploid origins are often limited to dozens of events even when parental species are sympatric or parapatric. Understanding the details of the evolutionary history of polyploids may facilitate monitoring and prediction of the dynamics of natural populations and species diversity as well as manipulation of the genetic diversity of agriculturally important crops.
